# Engaging Communities to Prevent Underage Drinking

**Published:** 2011

**Authors:** Abigail A. Fagan, J. David Hawkins, Richard F. Catalano

**Keywords:** Alcohol and other drug misuse, underage drinking, adolescent, risk factors, individual risk factors, risk and protective factors, environmental risk factors, prevention, prevention intervention, community-based prevention, school-based prevention, prevention program, curriculum, community coalition

## Abstract

Community-based efforts offer broad potential for achieving population-level reductions in alcohol misuse among youth and young adults. A common feature of successful community strategies is reliance on local coalitions to select and fully implement preventive interventions that have been shown to be effective in changing factors that influence risk of youth engaging in alcohol use, including both proximal influences and structural and/or environmental factors related to alcohol use. Inclusion of a universal, school-based prevention curriculum in the larger community-based effort is associated with the reduction of alcohol use by youth younger than 18 years of age and can help reach large numbers of youth with effective alcohol misuse prevention.

Research has identified multiple risk factors that increase the likelihood of alcohol use among youth and young adults. These conditions or experiences include individual characteristics (e.g., displaying aggression at a young age or believing that alcohol use is not harmful), peer influences (e.g., having friends who use alcohol or who believe that alcohol use is acceptable), family experiences (e.g., heavy alcohol use by parents or siblings, or inadequate parental supervision), school factors (e.g., academic failure, or having a low commitment to school or education), and neighborhood experiences (e.g., availability of alcohol to youth, or community norms that are permissive of youth alcohol use) (Durlak 1998; Hawkins et al. 1992; Pentz 1998). Protective or promotive factors that ameliorate the negative influences of risk factors or directly reduce the likelihood of alcohol use among young people also exist in all areas of people’s lives. They include, for example, being attached to others who do not abuse alcohol, having a resilient temperament, or holding clear standards against the use of alcohol before one is of legal age (Pollard et al. 1999; Werner 1993).

Prevention efforts aimed at reducing rates of alcohol use typically do so by seeking to minimize the target population’s exposure to harmful risk factors and/or enhance protective/promotive factors (Coie et al. 1993; Munoz et al. 1996). Focusing prevention efforts on youth offers particularly great potential, because the early onset of drinking has been associated with an increased likelihood of alcohol dependence later in life (Hingson et al. 2006). Although many prevention efforts have been found to reduce tobacco, alcohol, and other drug use (Hawkins et al. 1995; National Research Council and Institute of Medicine 2009; Spoth et al. 2008), these strategies often are limited by addressing risk and protective factors in just one socialization domain. Thus, most of these efforts focus only on the most direct (i.e., proximal) causes of alcohol use, such as the availability of alcohol or peer or family influences, rather than targeting the complex contexts in which youth and young adults live. This narrow focus may reduce the overall impact and long-term effectiveness of alcohol-abuse prevention strategies, both because multiple factors affect alcohol use and because the effectiveness of any intervention likely is compromised if the environment in which people live is unfavorable to or does not support intervention goals and activities (Flay 2000; Wagenaar and Perry 1994).

One at least equally promising strategy for affecting rates of alcohol use, abuse, and dependence (not just among youth and young adults) centers on community-based efforts. Such approaches rely on multiple strategies intended to change a variety of factors that place individuals at risk for engaging in alcohol misuse (Pentz 1998; Wandersman and Florin 2003). Most of these efforts seek to alter not only proximal influences, but also the long-term, structural, and environmental influences associated with alcohol abuse and dependence, which increases their potential to make a significant and long-lasting impact (Wagenaar et al. 1994). By saturating the environment with prevention strategies and messages, community-based efforts aim to reach many individuals, which may allow them to achieve population-level reductions in alcohol misuse.

Another potential advantage of community-based strategies is their reliance on members of the local community to plan, implement, and monitor prevention activities, usually via coalitions made up of stakeholders from diverse organizations and backgrounds. By actively involving the community in the prevention effort, these approaches may enhance community buy-in for prevention activities and may help to ensure that services are a good fit with local needs, resources, and norms (Hawkins et al. 2002; Stevenson and Mitchell, 2003; Wandersman et al. 2003; Woolf 2008). The levels of risk and protective/promotive factors vary across communities, and measures most needed in one community to reduce youth alcohol use may not be needed in another community (Hawkins et al. 2002; Reiss and Price 1996). Thus, prevention efforts that are based upon assessing local needs (i.e., risk and protective/promotive factors faced by those in the community) and implementing prevention strategies that are best suited to address these needs may be more effective than implementing a single prevention program across many communities. Community mobilization also may allow for effective pooling of information and resources across agencies and individuals, minimizing duplication of services, and potentially offering more cost-effective services that can be implemented better and are more likely to be sustained.

After defining what exactly community mobilization implies, this article explores what community-based strategies work to reduce alcohol use and misuse among youth and the role of school-based interventions in the context of community-level efforts. Finally, the article looks at the challenges associated with the successful implementation of community-based programs to prevent youth alcohol use.

## What Is Community Mobilization to Prevent Alcohol Misuse?

Existing community-based alcohol abuse prevention efforts are tailored to local circumstances, which makes it difficult to identify the specific components that define this type of approach. Nonetheless, community mobilization efforts have in common the goal of reducing alcohol misuse by changing the larger environment, using approaches that are owned and operated by the local community (Wandersman et al. 2003). Most programs rely on coalitions of community stakeholders to collaboratively plan and coordinate prevention activities. In some cases, coalitions focus on implementing, in a coordinated fashion, multiple, discrete prevention programs and practices that seek to decrease elevated risk factors and enhance depressed protective/promotive factors related to alcohol use (Hawkins et al. 2002). Other efforts specifically focus on transforming the environment via changes in local ordinances, norms, and policies related to alcohol. These latter efforts target a more limited number of risk factors, particularly community norms and laws related to alcohol use, the availability of alcohol, and individual attitudes favorable to alcohol use (Pentz 2000). Some community-based efforts rely on a combination of these strategies.

## What Community-Based Strategies Work to Reduce Alcohol Misuse Among Youth?

The findings presented in this article are based upon a comprehensive review of evaluations conducted in the United States that involved the implementation of a substantial, community-based prevention initiative aimed at reducing alcohol and other drug (AOD) use among minors (i.e., adolescents and young adults age 20 or younger). Projects were included in the review if they met the following criteria:

- They were evaluated using a well-conducted quasi-experimental or true experimental design that involved, at a minimum, one intervention group (implementing the strategy) and one comparison group.
- Data on alcohol use outcomes were collected at least twice during the research project (e.g., before and after the intervention was conducted).
- There were no significant threats to the validity and reliability of the study, as determined by the first two authors of this review.

Although many studies were reviewed, only nine community-based initiatives demonstrated reduced rates of alcohol use or alcohol availability among youth and young adults according to the above criteria (see the table). It is notable that several of these strategies affected not only alcohol use but also the use of tobacco and, in some cases, other illicit drugs. The table briefly describes each program, the population in which the intervention was evaluated, and the program’s significant effects in reducing AOD use.

The findings allow the following conclusions. First, a common feature of successful community-based prevention approaches is reliance on local coalitions to select effective preventive interventions and implement them with fidelity. Second, the inclusion of a universal, school-based drug prevention curriculum as part of the larger community initiative is associated with reductions in alcohol use among middle- and high-school students. Third, environmental strategies focused on changing local laws, norms, and policies related to alcohol access and use do not appear to reduce alcohol use among adolescents younger than age 18 when implemented independently of other community-based strategies. However, they have been part of successful multicomponent interventions and, when implemented on their own, have reduced the availability of alcohol in communities and lowered the rate of drunk-driving arrests among young adults.

### Reliance on Community Coalitions

All of the community-based initiatives listed in the table relied on local coalitions to plan and implement prevention activities. This observation indicates that to be successful, community efforts must ensure the presence of active, broad-based groups of individuals who believe it is possible to prevent youth AOD use and who are willing to engage in collaborative prevention activities. Although coalitions vary in their structures, sizes, goals, and activities, a defining feature of such groups is their focus on facilitating desired changes through collaborative action. Although the specific members of a coalition may vary depending on the focus of the group, coalitions usually seek to be broad based and to unite diverse stakeholders and key leaders from key agencies and sectors of the community. For example, coalitions focused on preventing alcohol use by youth may include representatives from law enforcement, local government, schools, health and human service agencies, youth service groups, business, religious groups, youth, and parents. The coalitions typically are formed around a common vision that inspires and motivates their actions. By working together to bring about change, they allow intervention approaches to be tailored to local needs, as identified by coalition members. They also increase political alliances, foster communication among community members, and coordinate human and financial resources (Hawkins et al. 2002; Pentz 2000; Wandersman et al. 2003).

Although coalitions are a common element of effective community-based prevention, not all coalition efforts have produced significant changes in alcohol use. Some coalition initiatives have failed to reduce rates of AOD use among youth and adolescents, even when they were well funded and members were well intentioned and willing to make changes. Evaluations of two coalition efforts—the Fighting Back (Hallfors and Godette 2002) and Community Partnership (Yin et al. 1997) initiatives—found that both failed to bring about changes in youth AOD use. The evaluations indicated that the coalitions involved in these projects had insufficient guidance in how to enact prevention strategies, varied widely in the nature and amount of prevention services provided, and largely relied on locally created prevention strategies that likely had not been previously evaluated for effectiveness in reducing AOD use. These studies suggest that the mere presence of an active, well-intentioned coalition is not enough to prevent AOD use. In other words, simply gathering local stakeholders and asking them to collaborate to do their best to solve local drug problems or prevent underage drinking does not produce desired changes.

Instead, the evidence suggests that in order to be successful, coalitions must ensure the following (Hallfors et al. 2002):

- They must have clearly defined, focused, and manageable goals;
- They must have adequate planning time;
- Prevention decisions must be based on empirical data about what needs to change in the community and on evidence from scientifically valid studies of what has worked to address those needs;
- They must implement prevention policies, practices, and programs that have been tested and shown to be effective; and
- They must carefully monitor prevention activities to ensure implementation quality.

One prevention system that exemplifies these principles is Communities That Care (CTC), which has been found to reduce the initiation and prevalence of youth alcohol use communitywide (Feinberg et al. 2007; Hawkins et al. 2009). CTC provides proactive training and technical assistance to community coalitions to ensure that they select and implement prevention strategies that previously have been demonstrated to be effective in reducing youth AOD use. The CTC model involves a structured and guided intervention process involving five phases in which coalitions (1) assess community readiness to undertake collaborative prevention efforts, (2) form a diverse and representative prevention coalition, (3) use epidemiologic data to assess prevention needs, (4) select evidence-based prevention policies and programs that target these needs, and (5) implement the new policies and programs with monitoring to ensure fidelity and evaluation to ensure that goals are being met. The coalitions are structured, ideally with a chair person, cochairs, and workgroups; employ at least a half-time coordinator; and are broad based. The prevention activities chosen and implemented can take place in a variety of settings and may target individual, family, school, peer, and/or community risk and protective/promotive factors related to youth AOD use. They are selected by the community coalitions from a menu of options that only includes policies and programs that have been shown in at least one study using a high-quality research design to significantly change risk and protective factors and reduce rates of AOD use (Hawkins and Catalano 1992; Hawkins et al. 2002).

Several evaluations of the CTC coalition model have been conducted, including a randomized trial involving 24 communities in 7 States that were randomly assigned to either implement the CTC system (*n* = 12) or serve as control communities (*n* = 12) (Hawkins et al. 2008). The intervention sites received training in the CTC model, proactive and intensive technical assistance, and funding for 5 years to plan and implement their chosen prevention strategies. This study found that after 4 years of the intervention, students in the CTC communities had lower rates of AOD use compared with students in control communities. They were less likely to initiate cigarette, alcohol, and smokeless tobacco use as well as delinquent behavior by the eighth grade. In addition, eighth-grade students in the intervention communities reported significantly lower rates of drinking, binge drinking, and smokeless tobacco use in the past month, as well as delinquent behavior in the past year, compared with students in the control communities (Hawkins et al. 2009).

These results indicate that when local community coalitions are provided with proactive training and technical assistance, have clear goals and guidelines, and ensure effective implementation of prevention strategies that have prior evidence of effectiveness, they have the potential to significantly reduce alcohol and tobacco use as well as delinquent behavior communitywide. Moreover, the findings indicate that coalitions may enact a variety of prevention policies and programs targeting a range of different risk and protective factors and still be successful, as long as their efforts focus on using methods that have been demonstrated to be effective and ensure that prevention activities are carefully implemented, monitored, and coordinated.

### Inclusion of School-Based Curricula in Community-Based Efforts

Implementation of universal, school-based drug prevention curricula as part of the larger community effort appears to predict reduced rates of AOD use among middle- and high-school students. All of the initiatives listed in the table that were effective in preventing or reducing alcohol use among those younger than age 18 involved the implementation of a school-based curriculum. Although neither the CTC prevention system nor the Kentucky Incentives for Prevention initiative (Collins et al. 2007) requires the use of school-based curricula, all of the coalitions involved in the randomized CTC evaluation (Hawkins et al. 2009), and all but one of the 19 coalitions evaluated in Kentucky, implemented a school curriculum to target particular risk factors whose influence in the community was considered too high or protective factors whose influence was considered too low by local coalitions.

The other community-based prevention initiatives listed in the table that reduced alcohol use among those younger than age 18 involved implementation of a particular school curriculum offered to students in conjunction with coalition-led efforts to change community-level risk factors related to drug use. The latter efforts typically attempted to change community norms and local ordinances related to alcohol use and availability. An evaluation of the Project Northland Program in Minnesota (Perry et al. 2002), for example, demonstrated reduced rates of alcohol use in communities that implemented a multiyear school curriculum and modified local policies and practices associated with youth alcohol use. The school program focused on altering student views regarding the acceptability of alcohol use, improving student skills in refusing drug offers, and fostering parent/child communication about alcohol use through homework assignments and information mailed to parents. Environmentally focused strategies included increased identification checks by retail liquor establishments and legal consequences for selling alcohol to minors. The evaluation of Project Northland found that after receiving services in both middle and high school, students in the intervention communities had lower rates of binge drinking (i.e., drinking five or more alcoholic beverages on one occasion) compared with students in control communities. In addition, retail establishments were less likely to sell alcohol to minors in intervention than in control communities (Perry et al. 2002).

A similar combination of activities was advocated in the Midwestern Prevention Project (MPP). This program involved the implementation of a 2-year middle-school curriculum to promote students’ drug resistance skills, along with parent education, media campaigns to reinforce antidrug messages throughout the community, and local policy changes to reduce demand and supply of drugs. When implemented in schools in Kansas City, the MPP demonstrated reductions in past-month smoking and alcohol use for students receiving the intervention compared with students in control schools (Pentz et al. 1989).

In Project SixTeen, small communities in Oregon implemented a five-session, school-based program aimed at reducing youth tobacco use, along with media campaigns and responsible beverage training for alcohol retail outlets. The evaluation showed a significant reduction in past-week smoking and marijuana use for seventh- and ninth-grade students in intervention communities compared with control communities; similarly, alcohol use was reduced among ninth graders (Biglan et al. 2000).

These studies indicate that the inclusion of school-based prevention programs in comprehensive, coalition-led, community-based initiatives can contribute to reductions in alcohol use among adolescents. Currently, most schools in the United States provide some type of drug-prevention programming to students. However, not all school districts implement strategies that have evidence of effectiveness, even though the Safe and Drug-Free School (SDFS) legislation mandates the use of effective substance-use prevention curricula. Inclusion of school-based programs in larger community prevention initiatives provides multiple advantages, including the ability to reach a large proportion of the youth population and thus increase the potential of achieving community-level changes in desired outcomes. Community coalitions can help school districts fulfill the SDFS mandate by helping them identify and adopt effective strategies and by helping to ensure that the new programs are well suited to addressing the needs of local students. In addition, coalitions can partner with schools to find the needed resources to initiate and sustain new effective prevention strategies and can help oversee the implementation of new strategies to ensure quality. To promote successful partnerships, coalitions should ensure that school personnel, including administrators (e.g., superintendents and principals) and staff (e.g., teachers and counselors), are actively involved in the decision making process and prevention efforts from the beginning of the initiative (Fagan et al. 2009).

### Targeting Environmental Risk Factors for Substance Use

The initiatives just described combined the implementation of school curricula with community mobilization efforts that target environmental risk factors in order to reduce the availability of and demand for alcohol. Such efforts include changes in community-level policies, practices, and norms, such as increasing alcohol pricing, creating drug-free zones, limiting alcohol sales in venues easily accessible to youth, requiring keg registrations, and increasing the use or severity of community laws related to alcohol use by minors or adults (Pentz 2000; Wagenaar et al. 1994). Changes in community practices also may involve responsible beverage service training—that is, educating merchants about the negative consequences of providing alcohol to minors or serving intoxicated patrons, encouraging identification checks, and ensuring that merchants who violate rules are appropriately sanctioned (Holder 2000). Media campaigns may also be used in conjunction with these activities to educate the public about the negative effects of alcohol use, increase support for drug prevention, and counter norms favorable to alcohol use. Such media campaigns increase public awareness by saturating the community with print, radio, and television advertisements; mailing informational fliers to businesses or homes; or holding community forums to discuss alcohol-use issues.

Evidence is mixed regarding the effectiveness of these types of environmentally focused prevention strategies. As discussed in the previous section, when offered in conjunction with school-based prevention curricula, these prevention strategies seem to be effective in reducing rates of adolescent alcohol use. However, efforts that focus exclusively on changing environmental risk factors at the local level, without also targeting more proximal risk factors related to alcohol use, have not been associated with reductions in alcohol use among youth under age 18. An evaluation of Communities Mobilizing for Change on Alcohol (CMCA) found no statistically significant changes (i.e., p<.05 using two-tailed test of significance) in alcohol or drug use among 12th-grade students or 18- to 20-year-olds in communities implementing CMCA compared with those in control communities (Wagenaar et al. 2000*b*). In this project, community coalitions coordinated a variety of activities aimed at limiting alcohol sales to minors, increasing enforcement of underage drinking laws, and changing alcohol policies at community events, as well as increasing public attention about problems associated with underage drinking. Although rates of alcohol use by youth were not significantly changed by the intervention, the evaluation did show that 18- to 20-year-olds from intervention sites were significantly less likely to provide alcohol to minors (Wagenaar et al. 2000*a*, *b*).

The Community Trials Project used similar environmentally focused prevention strategies to reduce alcohol use and related risky behaviors. A quasi-experimental evaluation of this program in six communities indicated significantly fewer alcohol-related automobile crashes in intervention communities than in control communities (Holder et al. 2000). Among adults (those age 18 or older), a greater proportion of those in communities implementing the program reported having one or more drinks in the past year versus those in comparison communities. However, among those who reported any drinking, adults in intervention sites had lower rates of self-reported heavy drinking and drunk driving (Holder et al. 2000). Although there were fewer sales to minors by alcohol sales establishments in intervention versus comparison sites (Grube 1997), none of the evaluations of the Community Trials Project have found significant reductions in drinking among youth under age 18 in intervention versus comparison sites.

The available evidence indicates that these types of community-based, environmentally focused strategies are effective in reducing alcohol use among those under age 18 only when offered in conjunction with effective school curricula. However, few evaluations have been conducted of community-based prevention efforts that rely solely on changing community policies, practices, and norms, and more research is needed to assess the impact of environmental strategies when used independently and when combined with other types of prevention strategies.

## Challenges Associated With Community Mobilization Efforts

There is much public support for community mobilization efforts that seek to reduce substance use, particularly by youth and young adults, and many communities have coalitions in place to coordinate local prevention strategies. However, implementing, evaluating, and sustaining such efforts can be challenging. For example, it often is difficult to recruit, engage, and ensure collaboration among community members from diverse backgrounds who may have different skills, needs, resources, and ideas about what is needed to prevent AOD use (Merzel and D’Afflitti 2003; Quinby et al. 2008; Stith et al. 2006). Furthermore, compared with single prevention programs, community-level strategies likely are costlier to implement and evaluate because they entail more components and require longer-term interventions to achieve community-wide outcomes (Merzel et al. 2003). It also can be difficult to define community boundaries, gain support for participation in a research study from key leaders and stakeholders, and measure processes and outcomes that may vary across communities (Stith et al. 2006; Wandersman et al. 2003). Finally, community-based prevention strategies are intended to be owned and operated by the community, which can create tension between local practitioners and scientists who may differ in their ideas about what is most needed to prevent alcohol misuse (Holder et al. 1997; Hyndman et al. 1992; Merzel et al. 2003).

The many challenges related to the implementation of community-based prevention efforts likely are responsible for the relatively small number of interventions that have demonstrated evidence of success (see the table). In addition, evaluations of some community prevention programs have failed to demonstrate significant effects on alcohol use, sometimes because of problems related to program implementation and intensity. For example, the initial evaluation of Project Northland in Minnesota indicated that the original 3-year intervention, which was implemented in middle schools, was insufficient to lead to sustained effects on alcohol use. Therefore, additional services were added in high schools, which reduced rates of alcohol use through grade 12 (Perry et al. 2002). A replication of this extended program in Chicago, Illinois, however, failed to produce positive effects, which led the evaluators to recommend that in lower-income, urban populations, where problems other than youth alcohol use (e.g., gangs, violence, and housing) may take precedence, longer-term and more intense community-based strategies may be needed to bring about change (Komro et al. 2008).

The Project Northland replication in Chicago and other evaluations have noted that implementation challenges, such as difficulties in engaging community members in the initiative and challenges in moving from planning to action, may compromise the ability of community-based efforts to produce significant effects. On the other hand, evaluations of the CTC prevention system have shown that communities can successfully mobilize volunteers, create high-functioning and goal-driven coalitions, and ensure high-quality implementation of prevention strategies that target salient risk and protective factors (Quinby et al. 2008). One factor that increases the likelihood of success is the provision of proactive and high-quality training and technical assistance from system developers to the community coalitions (Feinberg et al. 2008). In the absence of such training and technical assistance, common implementation challenges are likely to threaten implementation and the likelihood of realizing desired reductions in youth alcohol use.

Research also has indicated that communities that rely on prevention-focused coalitions, as in the CTC model, can successfully sustain the implementation of tested and effective programs, despite the human and financial costs associated with these efforts. An evaluation of 110 CTC coalitions in Pennsylvania (Feinberg et al. 2008) indicated that nearly all coalitions (91 percent), which still were operating after the State discontinued funding for CTC activities, continued to implement effective programs. In fact, on average, the coalitions were able to fund their program and coalition activities at levels exceeding those initially provided by the State. Funding success was positively associated with having a well-functioning coalition, adhering to the CTC model, and planning for sustainability. These findings reinforce the importance of utilizing broad-based coalitions to plan, implement, and sustain prevention activities in communities.

Identifying the cost-effectiveness of community-based prevention initiatives also is important. Although the review presented here identified nine community-based strategies with evidence of effectiveness in reducing alcohol use and availability among minors, only two of these interventions have been rigorously evaluated for cost-effectiveness. In both cases, the analyses demonstrated fiscal savings. According to the Washington State Institute for Public Policy (Aos et al. 2004), every dollar spent on Project Northland in Minnesota resulted in savings of $2.45 in later treatment, morbidity, mortality, and criminal justice costs; similarly, the MPP produced savings of $1.27 per dollar spent. Because cost is a major factor influencing community decisions to adopt new programs, information on financial benefits may help to increase the dissemination of community-based prevention strategies, their long-term sustainability, and ultimately their potential to substantially reduce rates of alcohol use among young people.

In summary, this review clearly has shown that community-based efforts can reduce alcohol use and misuse among youth. A common feature of successful community strategies is reliance on local coalitions to select and fully implement preventive interventions that have prior evidence of effectiveness in changing risk and protective or promotive factors related to alcohol use. Inclusion of a universal, school-based prevention curriculum in the larger community-based effort is associated with lower rates of drinking, binge drinking, and other drug use by those younger than 18. Focusing community-based prevention efforts on youth offers particularly great potential, because it not only lowers rates of alcohol use among minors but also reduces the likelihood of alcohol misuse and dependence later in life (Hingson et al. 2006).

## Figures and Tables

**Table:** Community Mobilization Strategies With Evidence of Effectiveness in Reducing the Use and/or Availability of Alcohol for Minors

Study	Description	Study Population	Significant Effects
Kentucky Incentives for Prevention (Collins et al. 2007)	Coalition-based prevention strategy targeting risk and protective factors related to drug use with effective programs conducted in schools and other community agencies	19 coalitions in Kentucky; 25,032 students in grades 8 and 10	Reduced smoking, drinking and binge drinking among 10th graders
Communities That Care (CTC) (Hawkins et al. 2009)	Coalition-based prevention strategy targeting elevated risk and depressed protective factors related to drug use with effective programs conducted in schools and other community agencies for peer review	24 communities in 7 States; 4,407 students in grade 5	Reduced the initiation of smoke-less tobacco, smoking, and alcohol Reduced past-month use of smokeless tobacco, alcohol, and binge drinking
Midwestern Prevention Project (Pentz et al. 1989)	Combines coalition-led community mobilization strategies with the implementation of school-based prevention curricula	42 schools in Kansas City; 5,065 students in grades 6 and 7	Reduced past-month smoking and drinking
Project SixTeen (Biglan et al. 2000)	Combines coalition-led community mobilization strategies with the implementation of school-based prevention curricula	16 communities in Oregon; 4,438 students in grades 7 and 9	Reduced smoking, drinking, and marijuana use
Project Northland (Perry et al. 2002)	Combines coalition-led community mobilization strategies with the implementation of school-based prevention curricula	24 school districts in Minnesota; 2,953 students in grade 6	Reduced binge drinking and alcohol sales to minors
Native American Project (Schinke et al. 2000)	Combines coalition-led community mobilization strategies with the implementation of school-based prevention curricula	27 tribal and public schools in the Midwest; 1,396 students in grades 3–5	Reduced smokeless tobacco, alcohol, and marijuana use
DARE Plus (Perry, Komro, Veblen-Mortenson et al. 2003)	Combines coalition-led community mobilization strategies with the implementation of school-based prevention curricula	24 schools in Minnesota; 7,261 students in grade 7	Reduced past-year and past-month smoking and drinking for boys and having ever been drunk for girls
Communities Mobilizing for Change on Alcohol (Wagenaar et al. 2000*a*, *b*)	Coalition-led activities seeking changes to community policies, practices, and norms related to alcohol use	15 school districts in Minnesota and Wisconsin; 4,506 students in grade 12, and 3,095 18-to 20-year-olds	Reduced the provision of alcohol to minors and arrests for drunk driving reported by 18-to 20-year-olds
Community Trials Project (Grube 1997; Holder et al. 2000)	Coalition-led activities seeking changes to community policies, practices, and norms related to alcohol use	6 communities in California and South Carolina	Reduced heavy drinking among adults, alcohol sales to minors, and alcohol-related car crashes

## References

[b1-arh-34-2-167] Aos S, Lieb R, Mayfield J (2004). Benefits and Costs of Prevention and Early Intervention Programs for Youth.

[b2-arh-34-2-167] Biglan A, Ary DV, Smolkowski K (2000). A randomised controlled trial of a community intervention to prevent adolescent tobacco use. Tobacco Control.

[b3-arh-34-2-167] Coie JD, Watt NF, West SG (1993). The science of prevention: A conceptual framework and some directions for a national research program. American Psychologist.

[b4-arh-34-2-167] Collins D, Johnson K, Becker BJ (2007). A meta-analysis of direct and mediating effects of community coalitions that implemented science-based substance abuse prevention interventions. Substance Use and Misuse.

[b5-arh-34-2-167] Durlak JA (1998). Common risk and protective factors in successful prevention programs. American Journal of Orthopsychiatry.

[b6-arh-34-2-167] Fagan AA, Brooke-Weiss B, Cady R, Hawkins JD (2009). If at first you don’t succeed... keep trying: Strategies to enhance coalition/school partnerships to implement school-based prevention programming. Australian & New Zealand Journal of Criminology.

[b7-arh-34-2-167] Feinberg ME, Bontempo DE, Greenberg MT (2008). Predictors and level of sustainability of community prevention coalitions. American Journal of Preventive Medicine.

[b8-arh-34-2-167] Feinberg ME, Greenberg MT, Osgood DW (2007). Effects of the Communities That Care model in Pennsylvania on youth risk and problem behaviors. Prevention Science.

[b9-arh-34-2-167] Feinberg ME, Ridenour TA, Greenberg MT (2008). The longitudinal effect of technical assistance dosage on the functioning of Communities That Care prevention boards in Pennsylvania. Journal of Primary Prevention.

[b10-arh-34-2-167] Flay BR (2000). Approaches to substance use prevention utilizing school curriculum plus social environment change. Addictive Behaviors.

[b11-arh-34-2-167] Grube JW (1997). Preventing sales of alcohol to minors: Results from a community trial. Addiction.

[b12-arh-34-2-167] Hallfors D, Godette D (2002). Will the “principles of effectiveness” improve prevention practice? Early findings from a diffusion study. Health Education Research.

[b13-arh-34-2-167] Hallfors D, Cho H, Livert D, Kadushin C (2002). Fighting back against substance use: Are community coalitions winning?. American Journal of Preventive Medicine.

[b14-arh-34-2-167] Hawkins JD, Catalano RF (1992). Communities That Care: Action for Drug Abuse Prevention.

[b15-arh-34-2-167] Hawkins JD, Arthur MW, Catalano RF, Tonry M, Farrington DP (1995). Preventing substance abuse. Crime and Justice: A Review of Research. Building a Safer Society: Strategic Approaches to Crime Prevention.

[b16-arh-34-2-167] Hawkins JD, Catalano RF, Arthur MW (2002). Promoting science-based prevention in communities. Addictive Behaviors.

[b17-arh-34-2-167] Hawkins JD, Catalano RF, Arthur MW (2008). Testing Communities That Care: The rationale, design and behavioral baseline equivalence of the Community Youth Development Study. Prevention Science.

[b18-arh-34-2-167] Hawkins JD, Catalano RF, Miller JY (1992). Risk and protective factors for alcohol and other drug problems in adolescence and early adulthood: Implications for substance abuse prevention. Psychological Bulletin.

[b19-arh-34-2-167] Hawkins JD, Oesterle S, Brown EC (2009). Results of a type 2 translational research trial to prevent adolescent drug use and delinquency: A test of Communities That Care. Archives of Pediatrics & Adolescent Medicine.

[b20-arh-34-2-167] Hingson RW, Heeren T, Winter MR (2006). Age at drinking onset and alcohol dependence: Age at onset, duration, and severity. Archives of Pediatrics & Adolescent Medicine.

[b21-arh-34-2-167] Holder HD (2000). Community prevention of alcohol problems. Addictive Behaviors.

[b22-arh-34-2-167] Holder HD, Gruenewald PJ, Ponicki WR (2000). Effect of community-based interventions on high-risk drinking and alcohol-related injuries. JAMA: Journal of the American Medical Association.

[b23-arh-34-2-167] Holder HD, Saltz RF, Grube JW (1997). Summing up: Lessons from a comprehensive community prevention trial. Addiction.

[b24-arh-34-2-167] Hyndman B, Giesbrecht N, Bernardi DR (1992). Preventing substance abuse through multicomponent community action research projects: Lessons learned from past experiences and challenges for future initiatives. Contemporary Drug Problems.

[b25-arh-34-2-167] Komro KA, Perry CL, Veblen-Mortenson S (2008). Outcomes from a randomized controlled trial of a multi-component alcohol use preventive intervention for urban youth: Project Northland Chicago. Addiction.

[b26-arh-34-2-167] Merzel C, D’Afflitti J (2003). Reconsidering community-based health promotion: Promise, performance, and potential. American Journal of Public Health.

[b27-arh-34-2-167] Munoz RF, Mrazek PJ, Haggerty RJ (1996). Institute of Medicine report on prevention of mental disorders: Summary and commentary. American Psychologist.

[b28-arh-34-2-167] National Research Council and Institute of Medicine (2009). Preventing Mental, Emotional, and Behavioral Disorders Among Young People: Progress and Possibilities. Committee on the Prevention of Mental Disorders and Substance Abuse Among Children, Youth, and Young Adults: Research Advances and Promising Interventions.

[b29-arh-34-2-167] Pentz MA, Sloboda Z, Hansen WB (1998). Preventing drug abuse through the community: Multicomponent programs make the difference. Putting Research to Work for the Community.

[b30-arh-34-2-167] Pentz MA (2000). Institutionalizing community-based prevention through policy change. Journal of Community Psychology.

[b31-arh-34-2-167] Pentz MA, Dwyer JH, MacKinnon DP (1989). A multicommunity trial for primary prevention of adolescent drug abuse: Effects on drug use prevalence. JAMA: Journal of the American Medical Association.

[b32-arh-34-2-167] Perry CL, Komro K, Veblen-Mortenson S (2003). A randomized controlled trial of the middle and junior high school D.A.R.E. and D.A.R.E. Plus programs. Archives of Pediatrics & Adolescent Medicine.

[b33-arh-34-2-167] Perry CL, Williams CL, Komro KA (2002). Project Northland: Long-term outcomes of community action to reduce adolescent alcohol use. Health Education Research.

[b34-arh-34-2-167] Pollard JA, Hawkins JD, Arthur MW (1999). Risk and protection: Are both necessary to understand diverse behavioral outcomes in adolescence?. Social Work Research.

[b35-arh-34-2-167] Quinby R, Fagan AA, Hanson K (2008). Installing the Communities That Care prevention system: Implementation progress and fidelity in a randomized controlled trial. Journal of Community Psychology.

[b36-arh-34-2-167] Reiss D, Price RH (1996). National research agenda for prevention research: The National Institute of Mental Health report. American Psychologist.

[b37-arh-34-2-167] Schinke SP, Tepavac L, Cole KC (2000). Preventing substance use among Native American youth: Three-year results. Addictive Behaviors.

[b38-arh-34-2-167] Spoth RL, Greenberg MT, Turrisi R (2008). Preventive interventions addressing underage drinking: State of the evidence and steps towards public health impact. Pediatrics.

[b39-arh-34-2-167] Stevenson JF, Mitchell RE (2003). Community-level collaboration for substance abuse prevention. Journal of Primary Prevention.

[b40-arh-34-2-167] Stith S, Pruitt I, Dees JE (2006). Implementing community-based prevention programming: A review of the literature. Journal of Primary Prevention.

[b41-arh-34-2-167] Wagenaar AC, Perry CL (1994). Community strategies for the reduction of youth drinking: Theory and application. Journal of Research on Adolescence.

[b42-arh-34-2-167] Wagenaar AC, Murray DM, Toomey TL (2000a). Communities Mobilizing for Change on Alcohol (CMCA): Effects of a randomized trial on arrests and traffic crashes. Addiction.

[b43-arh-34-2-167] Wagenaar AC, Murray DM, Gehan JP (2000b). Communities Mobilizing for Change on Alcohol: Outcomes from a randomized community trial. Journal of Studies on Alcohol.

[b44-arh-34-2-167] Wandersman A, Florin P (2003). Community intervention and effective prevention. American Psychologist.

[b45-arh-34-2-167] Werner EE (1993). Risk, resilience, and recovery: Perspectives from the Kauai longitudinal study. Development and Psychopathology.

[b46-arh-34-2-167] Woolf SH (2008). The power of prevention and what it requires. JAMA: Journal of the American Medical Association.

[b47-arh-34-2-167] Yin RK, Kaftarian SJ, Yu P, Jansen MA (1997). Outcomes from CSAP’s Community Partnership Program: Findings from the national cross-site evaluation. Evaluation and Program Planning.

